# The endoplasmic reticulum luminal Ca^2+^ regulates cardiac Ca^2+^ pump function

**DOI:** 10.1093/pnasnexus/pgaf045

**Published:** 2025-02-07

**Authors:** Elisa Bovo, Roman Nikolaienko, Daniel Kahn, L Michel Espinoza-Fonseca, Aleksey V Zima

**Affiliations:** Department of Cell and Molecular Physiology, Stritch School of Medicine, Loyola University Chicago, Maywood, IL 60153, USA; Department of Cell and Molecular Physiology, Stritch School of Medicine, Loyola University Chicago, Maywood, IL 60153, USA; Department of Cell and Molecular Physiology, Stritch School of Medicine, Loyola University Chicago, Maywood, IL 60153, USA; Division of Cardiovascular Medicine, Department of Internal Medicine, Center for Arrhythmia Research, University of Michigan, Ann Arbor, MI 48109, USA; Department of Cell and Molecular Physiology, Stritch School of Medicine, Loyola University Chicago, Maywood, IL 60153, USA

**Keywords:** calcium ATPase, intracellular calcium signaling, endoplasmic reticulum, fluorescent microscopy, calcium pump

## Abstract

The type 2a sarcoplasmic/endoplasmic reticulum Ca^2+^-ATPase (SERCA2a) plays a central role in Ca^2+^ signaling of cardiomyocytes. The speed at which SERCA2a pumps Ca^2+^ from the cytosol into the sarcoplasmic reticulum (SR) determines the diastolic relaxation rate. SERCA2a activity also sets SR Ca^2+^ load, which determines the amplitude of SR Ca^2+^ release and the systolic contraction strength. While SERCA2a controls the SR luminal [Ca^2+^] ([Ca^2+^]_SR_), less is known about how dynamic changes in [Ca^2+^]_SR_ affect SERCA2a function. By measuring the endoplasmic reticulum [Ca^2+^] ([Ca^2+^]_ER_) with the Ca^2+^ sensor R-CEPIA1er, we characterized the function of recombinant human and native mouse SERCA2a. We found that despite low endoplasmic reticulum (ER) Ca^2+^ gradient, SERCA2a-mediated Ca^2+^ transport was significantly slower at low [Ca^2+^]_ER_ than at intermediate [Ca^2+^]_ER_. It appears that certain [Ca^2+^]_ER_ is required for optimal SERCA2a Ca^2+^ transport. We tested whether negatively charged amino acids within the luminal loop between transmembrane helices M7 and M8 contribute to SERCA2a regulation by [Ca^2+^]_ER_. We found that the triple mutation E877L/D878L/E883L in the M7–M8 loop reduces SERCA2a Ca^2+^ transport particularly at intermediate [Ca^2+^]_ER_. Destabilizing the M7–M8 loop by breaking a disulfide bond between cysteines 875 and 887 abolished ER Ca^2+^ transport. Complementary molecular dynamics simulations showed that the triple mutant E877L/D878L/E883L stabilizes a Ca^2+^-bound E2 state of the pump, slowing down release of Ca^2+^ from the transport sites into the ER compared with the wild-type SERCA2a. These results revealed, for the first time, that SERCA2a Ca^2+^ transport is regulated by the luminal Ca^2+^ by interacting with the M7–M8 loop.

**Significance Statement**

The type 2a sarco/endoplasmic reticulum Ca^2+^-ATPase (SERCA2a) plays a central role in Ca^2+^ signaling of cardiomyocytes. Several pathologies, e.g. heart failure, are associated with impaired myocardial contraction due to reduced SERCA2a function. Therefore, understanding mechanisms of SERCA2a regulation is of great clinical importance. This study revealed a unique mechanism of SERCA2a regulation by the sarcoplasmic reticulum Ca^2+^. As a result of this regulation, SERCA2a works more efficiently at intermediate sarcoplasmic reticulum Ca^2+^ loads. Negatively charged amino acids E877, D878, and E883 in the luminal M7-M8 loop play a critical role in SERCA2a regulation by Ca^2+^. These findings provide new insight into mechanisms of abnormal Ca^2+^ regulation under conditions of chronically depleted the sarcoplasmic reticulum Ca^2+^ as reported in heart failure.

## Introduction

The type 2a sarcoplasmic/endoplasmic reticulum Ca^2+^-ATPase (SERCA2a) plays a central role in contraction and relaxation of the heart (1–3). The speed at which SERCA2a pumps Ca^2+^ from the cytosol into the sarcoplasmic/endoplasmic reticulum (SR/ER) is the main determinant of the rate of cardiac muscle relaxation during diastole. SERCA2a also sets the total amount of Ca^2+^ in the SR (i.e. SR Ca load), which determines the magnitude of SR Ca^2+^ release and the strength of systolic contraction (4). As SERCA2a plays an important role in cardiac Ca^2+^ regulation, impaired SERCA2a function has been reported in several pathological conditions, including heart failure (HF) (5–8). As a result, SERCA2a has attracted attention as a potential target for therapeutic interventions to improve heart function in HF patients. Although SERCA2a gene therapy showed promising results in initial preclinical studies (9, 10), it failed to reach primary and secondary endpoints in a following clinical trial on HF (11). While the exact reasons for this negative outcome are unclear, it might be that alteration of SERCA2a regulation, rather than the pump expression, plays a more important role in SR Ca^2+^ mishandling during cardiac pathologies. Therefore, there is a critical need to determine molecular mechanisms of SERCA2a regulation, mechanisms that may be the basis of alternative new therapies to enhance the Ca^2+^ pump function in the diseased heart.

Due to its critical role in cardiac function, SERCA2a is regulated by several different mechanisms to meet the changing body demand in the blood supply. The cardiac Ca^2+^ pump regulation is accomplished mainly through the interaction with the transmembrane peptide phospholamban (PLB). This peptide binds to SERCA2a and inhibits Ca^2+^ pump activity by lowering its apparent affinity for cytosolic Ca^2+^ (12). During β-adrenergic receptor stimulation, phosphorylation of PLB by protein kinase A relieves this inhibition, increasing the SERCA2a-mediated Ca^2+^ transport (13). This mechanism plays the key role in the acceleration of heart relaxation during diastole and increased contraction strength during systole under conditions of acute adrenergic stress (14, 15). Besides PLB regulation, SERCA2a also interacts with a wide array of small proteins, including histidine-rich protein, calreticulin, S100A, presenilin 1, and newly discovered micropeptides (16–19). Recently, we have provided evidence that SERCA2a can undergo dimerization, which increases both kinetic and thermodynamic efficacy of the pump (4).

Besides the activation of muscle contraction, Ca^2+^ within the SR lumen ([Ca^2+^]_SR_) plays an important role in the folding and regulation of luminal and transmembrane proteins (20–22). While SERCA2a Ca^2+^ transport controls [Ca^2+^]_SR_, less is known about how dynamic changes in [Ca^2+^]_SR_ affect SERCA2a function. The lack of selective and sensitive assays to measure the pump function in living cells limits our progress in understanding new mechanisms of SERCA2a regulation. In this study, we took advantage of our recently developed method to directly measure SR/ER Ca^2+^ uptake by recombinant or native SERCA2a (23). Using this approach, we discovered that SR/ER luminal Ca^2+^ plays an important role in the regulation of SERCA2a function by interacting with the luminal M7–M8 loop.

## Results

**Fig. 1.**
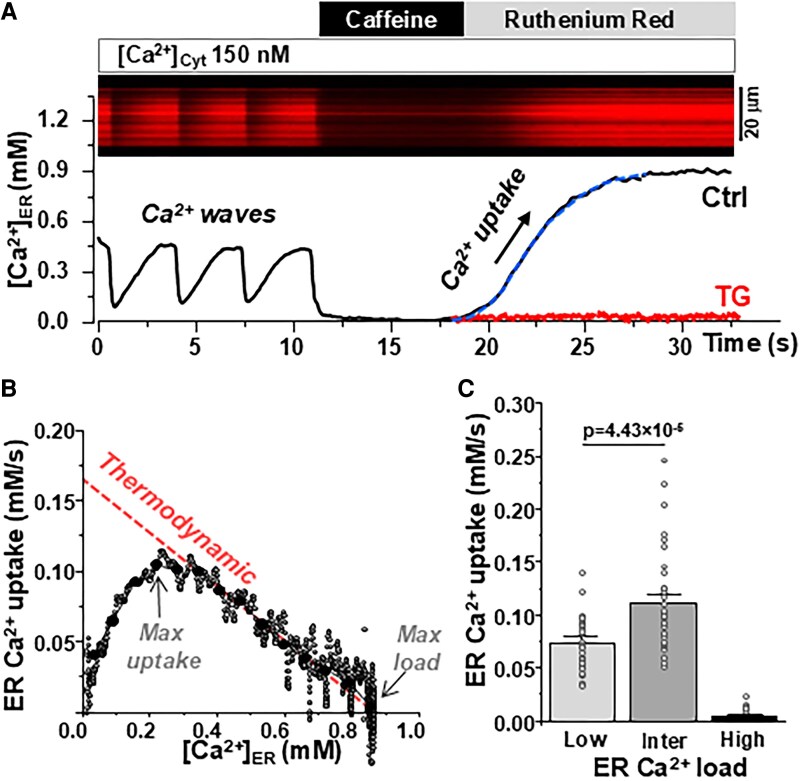
SERCA2a-mediated Ca^2+^ uptake as a function of [Ca^2+^]_ER_ in HEK239 cells expressing human SERCA2a. A) Experimental protocol to measure SERCA-mediated ER Ca^2+^ uptake. A line-scan image of R-CEPIA1er (top) and corresponding profile of changes in [Ca^2+^]_ER_ (bottom) were measured in control conditions, during RyR2 activation with 5 mM caffeine with following RyR2 inhibition with 15 μM ruthenium red. In the end of each experiment, the R-CEPIA1er signal was converted to [Ca^2+^] after measuring *F*_max_ in the presence 5 mM Ca^2+^ and 5 μM ionomycin and *F*_min_ in the presence of 5 mM caffeine. Black line (Ctrl)—control conditions. Red line (TG)—thapsigargin. B) For each individual cell, ER Ca^2+^ uptake was analyzed as the first derivative (d[Ca^2+^]_ER_/d*t*) and plotted as a function of ER Ca^2+^ load ([Ca^2+^]_ER_). C) The average results of SERCA2a-mediated Ca^2+^ uptake rate at different ER Ca^2+^ loads: low Ca^2+^ loads were within the range 0.05–0.10 mM; intermediate (inter) loads were within 0.2–0.4 mM and high loads were within 0.6–0.8 mM. The analysis is based on results from 40 cells.

**Fig. 2.**
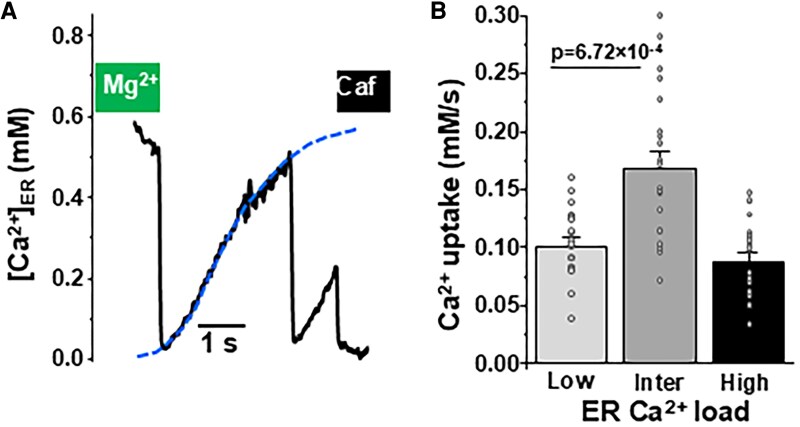
SERCA-mediated ER Ca^2+^ uptake during Ca^2+^ waves activated by relieving RyR2 inhibition. A) An example of Ca^2+^ waves recorded after relieving RyR2 inhibition by 10 mM cytosolic [Mg^2+^]. B) The average results of SERCA2a-mediated Ca^2+^ uptake rate during Ca^2+^ wave at different ER Ca^2+^ loads: low 0.05–0.10 mM; intermedium (inter) 0.2–0.4 mM and high 0.5–0.6 mM. The analysis is based on results from 20 cells.

To better understand the role of [Ca^2+^]_ER_ in the regulation of SERCA2a function, we used our newly developed method to directly measure ER Ca^2+^ transport in living cells (23, 25). [Ca^2+^]_ER_ was recorded with the genetically encoded ER Ca^2+^ sensor R-CEPIA1er in HEK293 cells expressing human SERCA2a. SERCA2a was labeled with the fluorescent tag mCerluean-M1 (mCer) to identify cells expressing the pump. The cells were also transfected with the ER Ca^2+^ release channel ryanodine receptor (RyR2), which enables pharmacological manipulation of ER Ca^2+^ load. The plasma membrane was permeabilized and the cells were placed in the potassium-based cytosol-like solution with 150 nM [Ca^2+^]. Co-expression of SERCA2a and RyR2 produced periodic Ca^2+^ waves due to spontaneous RyR2 activation followed by SERCA2a-mediated Ca^2+^ uptake (Fig. 1A). SERCA2a Ca^2+^ transport was measured as the ER Ca^2+^ reuptake rate after full ER Ca^2+^ depletion by the RyR agonist caffeine (5 mM). To avoid confounding effects of ER Ca^2+^ leak, RyR2 was inhibited by ruthenium red (15 μM) during the Ca^2+^ uptake phase (Fig. 1A). The first derivative of ER Ca^2+^ uptake (d[Ca^2+^]_ER_/d*t*) was plotted against the corresponding [Ca^2+^]_ER_ to define the maximum ER Ca^2+^ uptake rate and the maximum ER Ca^2+^ load (Fig. 1B). The maximal uptake rate is determined by the kinetics of the pump's catalytic cycle. The maximal load is determined by the pump's catalytic efficiency and the thermodynamic limit of ATP/ADP ratio. We found that at intermediate-high [Ca^2+^]_ER_ (>0.3 mM), the Ca^2+^ uptake rate was inversely proportional to [Ca^2+^]_ER_ (Fig. 1B, black circles). As expected, SERCA2a activity progressively slowed down as [Ca^2+^]_ER_ reached the maximum Ca^2+^ load (i.e. the pump's thermodynamic limit). Surprisingly, at low [Ca^2+^]_ER_ (≤0.3 mM), Ca^2+^ uptake did not follow the inverse relationship (red dashed line). Despite low ER Ca^2+^ gradient, SERCA2a Ca^2+^ transport was surprisingly slow at low ER Ca^2+^ loads. The deviation of experimental points from the predicted thermodynamic relationship at low ER Ca^2+^ loads can be explained if certain [Ca^2+^]_ER_ is required for optimal SERCA2a function. To define the sensitivity of SERCA2a to luminal Ca^2+^, we subtracted the experimental points from the thermodynamic relationship curve. This analysis revealed that an apparent affinity (*K_d_*) of the pump to luminal Ca^2+^ is ∼0.14 mM. Figure 1C summarizes the results of SERCA2a Ca^2+^ uptake rate at different ER Ca^2+^ loads. It appears that the ER Ca^2+^ uptake at intermediate [Ca^2+^]_ER_ loads (0.3–0.4 mM) is 70% higher than the uptake at low [Ca^2+^]_ER_ loads (0.05–0.2 mM): 0.12 ± 0.01 vs. 0.07 ± 0.01 mM/s (*n* = 40 cells). We tested whether the effect of luminal [Ca^2+^] on ER Ca^2+^ uptake is affected by higher cytosolic [Ca^2+^]. As for 150 nM, we observed similar bell-shape relationship between ER Ca^2+^ uptake and [Ca^2+^]_ER_ at 600 nM cytosolic [Ca^2+^] (Fig. S1). We also analyzed SERCA2a function during Ca^2+^ waves induced by RyR2 re-activation after relieving RyR2 inhibition by high cytosolic [Mg^2+^] (Fig. 2A). These waves occurred at high Ca^2+^ loads and terminated almost at 0 [Ca^2+^]_ER_, thus Ca^2+^ uptake could be studied over a wide range of [Ca^2+^]_ER_ without pharmacological interventions. This approach ruled out potential contamination by caffeine during the refilling phase. This analysis also revealed that ER Ca^2+^ uptake rate was faster at intermediate than low ER Ca^2+^ loads (Fig. 2B). These new findings nicely correlate with a previously published work, showing an initial decrease in SERCA Ca^2+^ uptake rate at depleted SR Ca^2+^ load followed by an increase in uptake in ventricular myocytes (26).

**Fig. 3.**
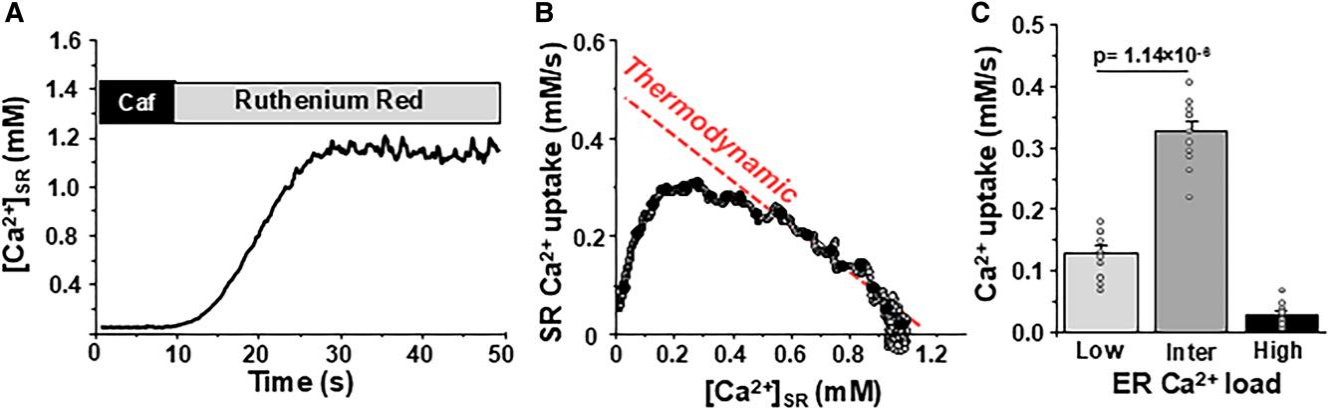
SERCA2a-mediated Ca^2+^ uptake as a function of [Ca^2+^]_SR_ in mouse ventricular myocytes. A) Profile of changes in [Ca^2+^]_SR_ during RyR2 activation with 5 mM caffeine with following RyR2 inhibition with 15 μM ruthenium red. The R-CEPIA1er signal was converted to [Ca^2+^] after measuring *F*_max_ in the presence 5 mM Ca^2+^ and 5 μM ionomycin and *F*_min_ in the presence of 5 mM caffeine. B) For each individual cell, SR Ca^2+^ uptake was analyzed as the first derivative (d[Ca^2+^]_ER_/d*t*) and plotted as a function of [Ca^2+^]_ER_. C) The average results of SERCA2a-mediated Ca^2+^ uptake rate at different SR Ca^2+^ loads: low 0.05–0.10 mM; intermedium (inter) 0.2–0.4 mM and high 0.6–0.8 mM. The analysis is based on results from 11 myocytes.

To study SERCA2a in its native environment, we used in vivo gene delivery of the Ca^2+^ sensor R-CEPIA1er cDNA with adenovirus in mice left ventricular wall (23). Six days after adenovirus injection, myocytes expressing R-CEPIA1er were isolated from the left ventricle. We have shown previously that myocytes expressing R-CEPIA1er show peaks of fluorescence at junctional SR, indicating proper localization of the Ca^2+^ sensor and regular SR Ca^2+^ release (23, 25). As in the HEK293 cell experiments, the same methodology was used to analyze SR Ca^2+^ uptake in permeabilized ventricular myocytes (Fig. 3A). This analysis revealed that native SERCA2a is also regulated by luminal Ca^2+^ with *K_d_* ∼0.22 mM (Fig. 3B). The ER Ca^2+^ uptake rate at intermediate [Ca^2+^]_ER_ loads (0.3–0.4 mM) was 0.33 ± 0.02 mM/s (*n* = 11 myocytes), whereas Ca^2+^ uptake at low [Ca^2+^]_ER_ loads (0.05–0.2 mM) was only 0.13 ± 0.01 (*n* = 11 myocytes). The contribution of the main luminal Ca^2+^ buffer calsequestrin was taken into account during the uptake analysis when free [Ca^2+^] was converted to total [Ca^2+^]_SR_ (27). We also used a different approach to evaluate SERCA2a regulation by luminal Ca^2+^ in myocytes. In these experiments, [Ca^2+^]_SR_ was measured in intact ventricular myocytes during regular electrical pacing (0.5 Hz). First, SR Ca^2+^ load was depleted with caffeine (5 mM). Then, caffeine was washed-out and electrical pacing was resumed. Consequently, SR Ca^2+^ load started gradually refilling after every consecutive action potential (Fig. S2). The analysis of SR Ca^2+^ uptake at different [Ca^2+^]_SR_ revealed that similar bell-shaped dependence as seen in permeabilized myocytes (Fig. 3). Collectively, these results reveal, for the first time, that luminal Ca^2+^ regulates recombinant and native SERCA2a function.

**Fig. 4.**
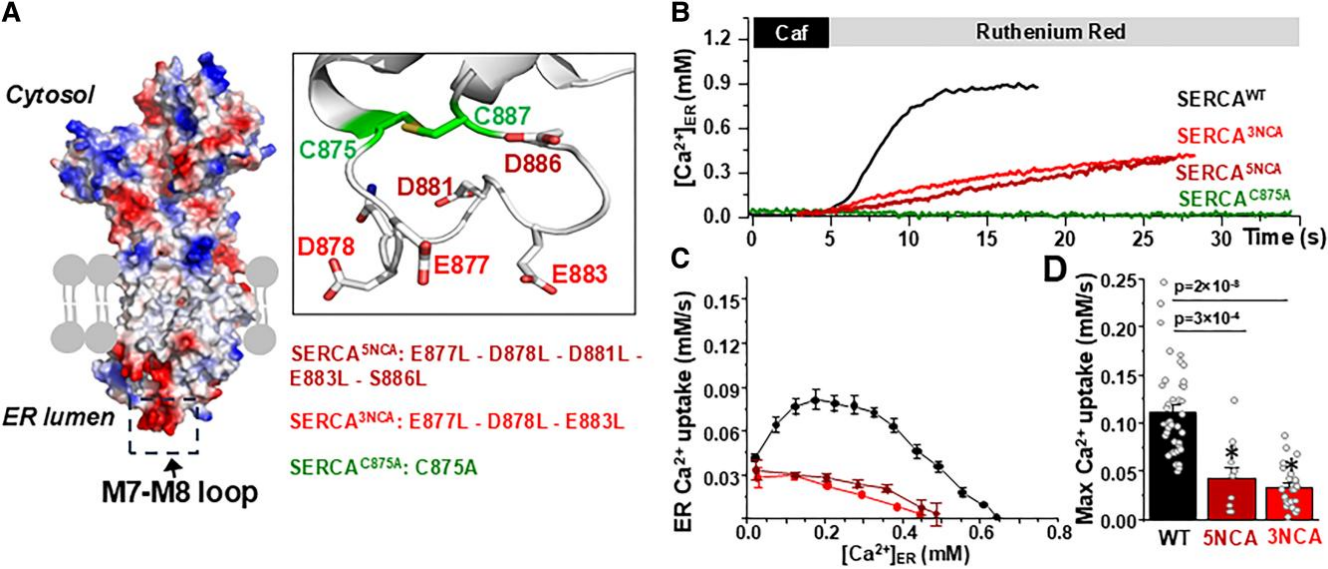
SERCA2a-mediated Ca^2+^ uptake as a function of [Ca^2+^]_ER_ in HEK239 cells expressing SERCA2a^WT^ and three M7-M8 SERCA2a mutants. A) Left: analysis of the electrostatic potential SERCA2a map. Negative charges are labeled in red. Positive charges are labeled in blue. Right: position of two cysteines (green) and five NCA (red) in the M7–M8 loop. The list of three SERCA2a mutants with NCAs and single cysteine mutations. B) Changes in [Ca^2+^]_SR_ during RyR2 activation with following RyR2 inhibition in HE293 cells expressing SERCA2a^WT^ and different SERCA2a mutants: SERCA2a^C875A^, SERCA2a^5NCA^, and SERCA2a^3NCA^. The R-CEPIA1er signal was converted to [Ca^2+^] after measuring *F*_max_ in the presence 5 mM Ca^2+^ and 5 μM ionomycin and *F*_min_ in the presence of 5 mM caffeine. C) ER Ca^2+^ uptake was analyzed as the first derivative (d[Ca^2+^]_ER_/d*t*) and plotted as a function of [Ca^2+^]_ER_. D) The average results of maximal ER Ca^2+^ uptake rate in HE293 cells expressing SERCA2a^WT^ and different SERCA2a mutants. The analysis is based on results from 40 cells for SERCA2a^WT^, 9 cells for SERCA2a^5NCA^ and 24 cells for SERCA2a^3NCA^.

SERCA2a contains 10 transmembrane helices (M1–M10). The luminal loops are short, with one exception: the loop connecting M7 and M8. This structural feature consists of 46 amino acids that protrude into the luminal space (Fig. 4A). Analysis of the previously solved SERCA2a crystal structure (28, 29) reveals a confluence of negative electric potential in this loop (Fig. 4A, left, marked by red), conferred by five negatively charged amino acids (NCA): E877, D878, D881, E883, and D886 (Fig. 4A, right). We hypothesized that the luminal Ca^2+^ interacting with NCA on the M7–M8 loop stabilizes the orientation of M7 and M8 relative to other helices, optimizing Ca^2+^ transport. First, we studied the role of these NCA by generating the mCer-SERCA2a^5NCA^ mutant where all NCA were mutated to leucine (E877L/D878L/D881L/E883L/D886L). These studies revealed that these mutations reduce SERCA2a Ca^2+^ transport, predominantly at intermediate-high [Ca^2+^]_ER_ (Fig. 4B and C). Interestingly, similar results were obtained by mutating only three NCA: E877L/D878L/E883L (Fig. 4B and C). To rule out the effect of difference in SERCA2a expression, the analysis was performed in cells with comparable the mCer signal: SERCA2a^WT^ 89 ± 7 AU (*n* = 39 cells), SERCA2a^5NCA^ 80 ± 11 AU (*n* = 14 cells), and SERCA2a^3NCA^ 78 ± 9 (*n* = 24 cells). This analysis suggests that NCA E877, D878, and E883 in the M7–M8 loop play a particularly important role in SERCA2a regulation by the luminal Ca^2+^.

The SERCA2a M7–M8 loop also contains two cysteines: C875 and C887 (Fig. 4A, right, marked by green). As the ER luminal redox potential is predominantly oxidized (30, 31), the loop structure is stabilized by a disulfide bond between these two cysteines. Indeed, the structural analyses that elucidated the pump catalytic cycle in atomic detail all harbor a disulfide bonds between these two cysteines (3, 32). We tested whether a disruption of the disulfide bond affects SERCA2a function and its luminal Ca^2+^ regulation. As only one cysteine mutation is required to abolish the disulfide bond formation, we designed the pump construct with cysteine 875 mutated to alanine (SERCA2a^C875A^). By analyzing an averaged mCer signal in each studied HEK293 cells expressing mCer-SERCA2a^WT^ and mCer-SERCA2a^C875A^, we found that the C875A mutation drastically reduced the SERCA2a expression level. Only 15% of the transfected cells expressed the C875 mutant. Furthermore, the analysis of cells expressing mCer revealed that the mutation reduced the pump expression by 41 ± 5% (*n* = 33 wild-type [WT] and *n* = 12 mutant cells). These results were confirmed by the western blot analysis (Fig. S3). Importantly, the C875A mutation completely depletes ER Ca^2+^ load due to dramatic inhibition of ER Ca^2+^ transport in cells expressing mCer tag (Fig. 4B, green), suggesting that the structural integrity of the M7–M8 loop is critical for the pump function.

**Fig. 5.**
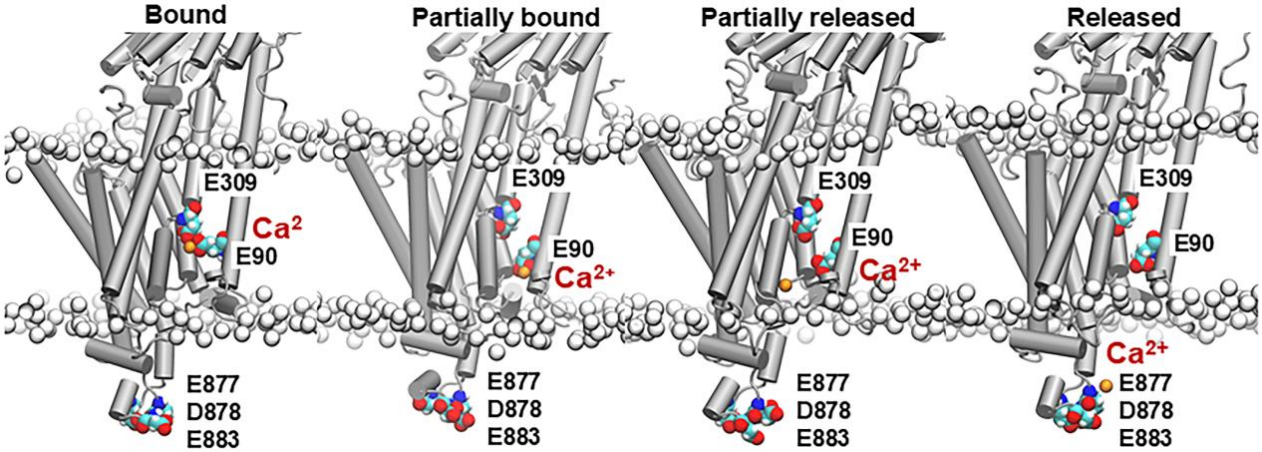
Representation of translocation of a single Ca^2+^ ion from the transport sites of SERCA2a into the SR lumen. Structures of the SERCA2a embedded in a lipid bilayer extracted from the MD trajectories of the WT SERCA bound to a single Ca^2+^ ion. The head groups of the lipid bilayer are shown as white spheres; SERCA2a is shown as a gray cartoon representation. Key SERCA2a residues and the Ca^2+^ ion are shown as a van der Waals representation. We note that this translocation mechanism is inhibited upon mutation of residues E877, D878, and E883 to leucine.

**Fig. 6.**
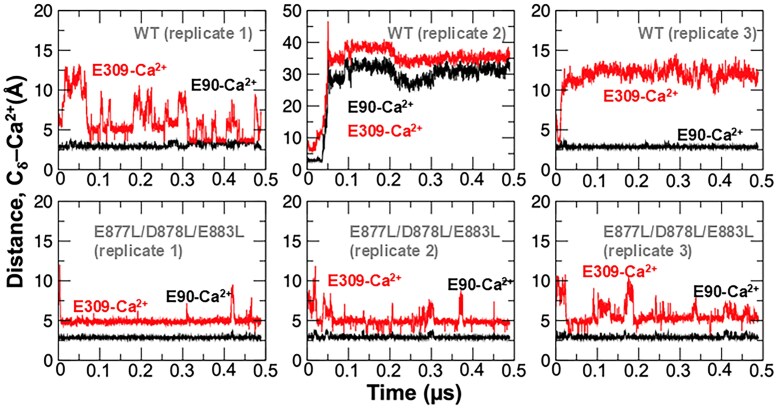
Distance evolution between residues E90 and E309 with the Ca^2+^ ion occluded in the transport sites in the WT and triple mutant SERCA2a. Distances were calculated from each independent MD trajectory between the carbon delta (Cδ) of residues E90 (black trace) or E309 (red trace) and the Ca^2+^ ion. For these simulations, we used the crystal structure of SERCA2a representing the state at which Ca^2+^ is released into the SR lumen (PDB: 3b9b). This structure, which was obtained in the absence of Ca^2+^, a single Mg^2+^ ion was resolved in the transport sites; we replaced this ion for Ca^2+^ as a starting point for performing the atomistic MD simulations. The plots show that in SERCA2a^WT^, Ca^2+^ partially (e.g. replicates 1 and 3) or fully (e.g. replicate 2) translocate into the luminal side of the lipid bilayer. Translocation is inhibited upon mutation of residues E877, D878, and E883 to leucine.

We used molecular dynamics simulations to establish the structural mechanism by which the triple mutation E877L/D878L/E883L affects SERCA2a Ca^2+^ transport. We focus on this mutant because its impaired pump function is not linked to issues with expression, as observed for the SERCA2a^C875A^ mutant. When analyzing the trajectories, we focused on the ability of Ca^2+^ to translocate from the transport sites to the luminal side of the lipid bilayer. Specifically, inhibited Ca^2+^ translocation occurs when Ca^2+^ interacts both with transport-site residues E90 and E309 (Fig. 5). Partial translocation of Ca^2+^ is defined by increasing distances between Ca^2+^ and SERCA residue E309, whereas full translocation occurs when the Ca^2+^ ion no longer interacts with either residues E90 or E309 (Fig. 5). Analysis of the trajectories showed that in the WT form of the pump, Ca^2+^ translocation occurs either partially (e.g. replicate 3, Fig. 6) or fully (e.g. replicate 2, Fig. 6) in the nanosecond timescale. Conversely, the simulations showed that the triple mutant E877L/D878L/E883L inhibits either partial or full Ca^2+^ translocation in the submicrosecond timescale (Fig. 6).

## Discussion

The aim of this study was to characterize the role of ER/SR luminal Ca^2+^ in the regulation of cardiac SERCA2a function. Using our new approach to directly measure ER Ca^2+^ transport in living cells, we found that despite low ER Ca^2+^ gradient SERCA2a-mediated Ca^2+^ uptake was unexpectedly slower at low ER Ca^2+^ loads than at intermediate ones. It appears that certain [Ca^2+^]_ER_ is necessary for optimal SERCA2a Ca^2+^ transport. The luminal Ca^2+^ regulation of SERCA2a was observed for native Ca^2+^ pump studied in mouse cardiomyocytes as well as for recombinant human SERCA2a expressed in HEK293 cells. The latter finding suggests that an interaction between the luminal Ca^2+^ and SERCA2a is direct and does not require additional auxiliary proteins. Further mutagenesis studies confirmed that Ca^2+^ interacts with several NCA on the luminal M7–M8 loop of the pump. Among five NCA in the M7–M8 loop, two glutamic acids 877 and 883 and one aspartic acid 878 play a particularly important role in the luminal Ca^2+^ regulation of SERCA2a function. Mutants of these amino acids to leucine reduced both the maximum ER Ca^2+^ uptake and load, suggesting that an interaction of Ca^2+^ with the M7–M8 loop improves SERCA2a function by increasing its catalytic and thermodynamic efficacy. Since the M8 helix is directly involved in Ca^2+^ transport (3), we hypothesized the orientation of its luminal end may be critical for optimal alignment of 30 Å distant residues further up in the helix where high-affinity Ca^2+^ transport occurs. Complementary molecular dynamics simulations showed that the triple mutant E877L/D878L/E883L inhibits translocation from the transport sites to the luminal side of the membrane. These simulations indicate that in the absence of these negatively charged residues, the pump is stabilized in a Ca^2+^-bound E2 intermediate state, slowing down release of Ca^2+^ from the transport sites into the ER compared with the WT pump. While there is consensus on the importance of negatively charged residues in SERCA for binding of Ca^2+^ ions from the cytosol (33–35), both the experiments and simulations demonstrate for the first time the role of acidic residues in the luminal side for translocation of Ca^2+^ into the ER.

As the luminal Ca^2+^ plays an important role in folding and regulation of ER proteins (20–22), consequently chronic [Ca^2+^]_ER_ depletion can trigger ER stress (36, 37). Recent evidence revealed that ER stress is involved in the development of various heart pathologies, including hypertrophy, ischemic heart diseases, and HF (38, 39). Moreover, chronic [Ca^2+^]_ER_ depletion has a significant impact on the luminal redox potential (25, 36, 40–42). As folding of luminal proteins is stabilized by disulfide bonds between neighboring cysteines, a unique oxidizing environment within the ER lumen plays a critical role in ER protein folding and regulation. ER stress is also associated with more reducing environments (i.e. reducing stress) due to alterations in function of several oxidoreductases and glutathione transferase (30, 31). Such changes in the luminal redox potential would affect the SR protein structure function by breaking luminal disulfide bonds. SERCA2a contains only two cysteines facing the ER lumen: C875 and C887. Interestingly, these cysteines are a part of the M7–M8 loop, a region that is involved in the luminal Ca^2+^ regulation of the pump. The structure analyses that elucidated the pump catalytic cycle in atomic detail all harbor disulfide bond between these two cysteines (3, 32). We generated the pump construct SERCA2a^C875A^ that abolishes the formation of disulfide bond within the M7–M8 loop. Expression of SERCA2a^C875A^ in HEK293 cells depleted ER Ca^2+^ load due to drastic reduction of the pump expression and the pump Ca^2+^ transport, suggesting an important role of the structural integrity of the M7–M8 loop for the pump expression and function. These findings are in good agreement with the previously published work showing that the formation of disulfide bond between the luminal C878 and C888 requires for SERCA1a function (43). Mutations of these cysteines possessed high ATPase activity, but without Ca^2+^ transport. It appears that the intact M7–M8 loop structure is critical for a proper coupling between ATPase activity and Ca^2+^ transport. However, another study on SERCA2b suggested that the formation of disulfide bond in the M7–M8 loop inhibits ER Ca^2+^ transport, and this mechanism is mediated by an interaction with calnexin and the ER oxidoreductase ERp57 (44). It appears that the significant differences in SERCA1a/2a and SERCA2b structure, such as an extra transmembrane domain in SERCA2b, might contribute to differences in the pump's redox regulation.

In conclusion, the results of this study revealed, for the first time, that SERCA2a function is regulated by the luminal Ca^2+^ by interacting with the M7–M8 loop. As a result of the luminal Ca^2+^ regulation, SERCA2a works more efficiently at the [Ca^2+^]_SR_ range >0.3 mM. This is the same range at which SR Ca^2+^ machinery operates on a beat-to-beat basis in healthy cardiac myocytes (45–47). We expect that this new mechanism of SERCA2a regulation plays a fundamental role in well-tuned SR Ca^2+^ signaling of the healthy heart. Any defects in the pump's luminal Ca^2+^ regulation would decrease the pump's catalytic and thermodynamic efficacy, reducing SR Ca^2+^ release and myocardial contraction. The identification of new molecular sites of SERCA2a regulation should widen the possibilities for new pharmacological and genetic approaches to improve the pump's function in various heart diseases.

## Materials and methods

### Vectors and adenovirus production and acquisition

pCMV R-CEPIA1er was a gift from Dr Masamitsu Iino (Addgene, USA). The vector encoding the human RyR2 cDNA fused to GFP at the N-terminus domain was kindly provided by Dr. Christopher George (University of Cardiff, UK). The vector encoding human WT SERCA2a (SERCA2a^WT^) cDNA was kindly provided by Dr David Thomas (University of Minnesota, USA). The SERCA2a cDNA was cloned into the mCerluean-M1 (mCer) modified plasmid (Addgene) using KpnI and NotI restriction enzymes, yielding SERCA2a fused to a modified Cerulean fluorescent protein (mCer) at the N-terminus. SERCA2a cDNA was also cloned into the inducible expression vector pcDNA5/FRT/TO for the generation of SERCA2a stable cell line. The sequences were all verified by single pass primer extension analysis (ACGT Inc., USA) (48). All SERCA2a mutants were generated using a site-directed mutagenesis kit (Q5-site-directed mutagenesis kit, NEB). Specific primers containing the mutations were used to amplify the whole plasmid containing the cerulean tagged SERCA2a gene. After verification of the mutagenesis by single pass analysis, the plasmids were amplified and used for experimentation. The R-CEPIA1er adenovirus was generated and provided by Dr Jody Martin, University of California in Davis.

### In vivo gene delivery of R-CEPIA1er

All animal husbandry and surgical procedures were carried out in accordance with the National Institutes of Health guide for the care and use of laboratory animals (49). All animal procedures take place in the Center for Translational Research and Education at Loyola University Chicago. Procedures are covered by Loyola IACUC protocols #23-027 (mice). C57BL/6J mice (eight of male and five of female animals, Jackson Laboratories) were maintained on a 12:12 h light–dark cycle and were given ad libitum food and water throughout the experiments. Eight- to 10-week-old mice, weighing 20–25 g, were used in the experiments. Mice were anesthetized with isoflurane at a dose of 1–3% with oxygen (0.5%). Artificial respiration was maintained with a rodent ventilator. The heart was exposed upon opening of the left pleural cavity by cutting the intercostal muscles and by positioning a retractor between the left third and fourth ribs. The R-CEPIA1er adenovirus (5 × 10^10^ particles/mL) was administered by direct injection in the left ventricular wall of the myocardium (6 spots, 8 μL/site) using a syringe fitted with a 29-G needle. After the injection, the chest was closed in layers. Mice were carefully monitored for postoperative recovery and given free access to the analgesic carprofen. The expression of the R-CEPIA1er transgene was optimal at 6 days postinjection.

### Ventricular myocytes isolation

C57BL/6J mice aged between 2 and 5 months were anesthetized using isoflurane (3%). Following thoracotomy, hearts were quickly excised and mounted on a Langendorff apparatus for retrograde perfusion with a solution containing collagenase, Liberase H. The ventricular myocytes were isolated from the left ventricle, as previously described (50–52). In brief, the left ventricle was excised from the heart, placed in stop buffer containing bovine serum albumin 1 mg/mL and cut into several pieces. Following gentle breakdown of the muscle to single cells, myocytes (∼0.1 mL) were pelleted by gravity and resuspended in low-Ca^2+^ Tyrode buffer (in mM: NaCl 140; KCl 4; CaCl_2_ 0.1; MgCl_2_ 1; glucose 10; HEPES 10; pH 7.4). [Ca^2+^] was gradually adjusted to 1 mM. Isolated cardiomyocytes were stored at room temperature (20 °C). All chemicals and reagents were purchased from Sigma-Aldrich (St Louis, MO, USA).

### Experimental protocol

A similar experimental protocol as described in (23) was used to study SERCA2a function. HEK293 cells at 60–80% confluency were transiently co-transfected with plasmids containing the cDNA of RyR2, SERCA2a (WT or mutant), and R-CEPIA1er. Experiments were conducted 48 h after transfection to obtain the optimal level of recombinant proteins expression. Flp-In T-Rex-293 stably expressing SERCA2a was co-transfected with plasmids containing the cDNA of RyR2 and R-CEPIA1er, using the same conditions for the HEK293 cells. In HEK293 cells and myocyte experiments, the surface membrane was permeabilized with saponin (0.005%). Experiments were conducted after washing out of saponin with an experimental solution (in mM): K-aspartate 100; KCl 15; KH_2_PO_4_ 5; MgATP 5; EGTA 0.35; CaCl_2_ 0.22; MgCl_2_ 0.75; HEPES 10; dextran (MW: 40,000) 2%; and pH 7.2. Free [Ca^2+^] and free [Mg^2+^] of this solution were 150 nM and 1 mM, correspondingly. Experimental solutions were replaced by using a fast local perfusion system. To estimate the rate of solution replacement, we measured the speed of Fluo-4 (potassium salt) entry-in and washout from permeabilized cells at similar experimental conditions as for [Ca^2+^] measurements. We found that the Fluo-4 signal reached maximum within half of a second (0.05 ± 0.01; *n* = 8) and can be fully washed from a cell in less than a second (0.85 ± 0.06 s; *n* = 8). To avoid caffeine contamination during ER Ca^2+^ uptake analysis, data points during the first second of the caffeine washout were excluded from the final analysis.

### Confocal microscopy

Expression of recombinant proteins and changes in the luminal ER [Ca^2+^] ([Ca]_ER_) were measured with laser scanning confocal microscopy (Radiance 2000 MP, Bio-Rad, UK) equipped with a ×40 oil-immersion objective lens (NA = 1.3). To measure the expression of SERCA2a, mCer was excited with the 458 nm line of the argon laser and signal collected at >485 nm. 2D images were collected at a speed of 6 ms/line. [Ca^2+^]_ER_ was recorded as changes in fluorescence intensity of the genetically encoded ER-targeted Ca^2+^ sensor R-CEPIA1er (23, 25, 53). R-CEPIA1er was excited with a 514 nm line of the argon laser, and signal was collected at >560 nm. Line scan images were collected at a speed of 20 ms/line. The R-CEPIA1er signal (*F*) in HEK293 cells and cardiomyocytes was converted to [Ca^2+^]_ER_ by the following formula: [Ca^2+^]_ER_ = *K_d_* × [(*F* − *F*_min_)/(*F*_max_ − *F*)]. *F*_max_ was recorded in 5 mM Ca^2+^ and 5 μM ionomycin and *F*_min_ was recorded after ER Ca^2+^ depletion with 5 mM caffeine. The *K_d_* (Ca^2+^ dissociation constant) of R-CEPIA1er was 609 μM (25). In cardiomyocytes, to account for the Ca^2+^ buffering power of SR Ca^2+^ binding protein calsequestrin. Total [Ca^2+^]_SR_ was calculated as: [Ca^2+^]_SR_ = *B*_max_/(1 + *K_d_*/[Ca^2+^]_SR_) + [Ca^2+^]_SR_, where *B*_max_ and *K_d_* of calsequestrin were 2,700 and 630 μM, respectively (27). SERCA-mediated Ca^2+^ uptake was calculated as the first derivative of [Ca^2+^]_ER/SR_ refilling (d[Ca^2+^]_ER/SR_/d*t*) after RyR2 inhibition with ruthenium red (15 μM) and tetracaine (1 mM). ER/SR Ca^2+^ uptake rate was plotted as a function of [Ca^2+^]_ER/SR_ to analyze maximum ER/SR Ca^2+^ uptake rate and maximum ER/SR Ca^2+^ load. All 2D images and line scan measurements for [Ca^2+^]_SR_ were analyzed with ImageJ software (NIH, USA).

### Preparation of the SERCA–lipid systems

We used the crystal structure of SERCA in the low Ca^2+^ affinity, E2 state (PDB: 3b9b) (3) as a starting model for the simulations of the WT and E877L/D878L/E883L SERCA (54). We chose this structure because it represents the state at which Ca^2+^ is translocated into the ER lumen. In this structure, the Mg^2+^ ion in the transport sites was replaced by Ca^2+^. We modeled transport-site residues Glu309 and Asp800 as unprotonated and residues Glu90, Glu771, and Glu908 as protonated. The complexes were embedded in a 130 × 130 Å bilayer of POPC lipids. The initial system was solvated using TIP3P water molecules with a margin of 20 Å in the *z*-axis between the edges of the periodic box and the cytosolic and luminal domains of SERCA, respectively. K^+^ and Cl^−^ ions were added to neutralize the system and to produce a KCl concentration of ∼100 mM. Preparation of the systems was done using the CHARMM-GUI web interface (55).

### Molecular dynamics simulations

We performed molecular simulations with AMBER20 on Tesla V100 GPUs (56) using the AMBER ff19SB force field (57). We maintained a temperature of 310 K with a Langevin thermostat and a pressure of 1.0 bar with the Monte Carlo barostat. We used the SHAKE algorithm to constrain all bonds involving hydrogens and allow a time step of 2 fs. We first performed 5,000 steps of steepest-descent energy minimization followed by equilibration using two 25-ps MD simulations using a canonical ensemble (NVT), one 25-ps MD simulation using an isothermal–isobaric ensemble (NPT), and two 500-ps MD simulations using the NPT ensemble. The equilibrated systems were used as a starting point to perform three independent 0.5-µs replicates of each system.

### Statistics

Data are presented as mean ± SEM of *n* measurements. Statistical comparisons between groups were performed with the Student's t test for unpaired datasets. Differences were considered statistically significant at *P* < 0.05. Statistical analysis and graphical representation of averaged data were carried out on OriginPro7.5 software (OriginLab, USA).

## Supplementary Material

pgaf045_Supplementary_Data

## Data Availability

The data supporting the findings of this article are available within in a public repository Figshare (doi: 10.6084/m9.figshare.28306787). The cryo-EM models of SERCA2a used in this study PDB ID: 5MPM (29) was obtained from Protein Data Bank. All cDNA expression vectors developed in this study are available on request from the corresponding author via their institutional contact details.

## Previous Presentation

Part of this work has been published in the conference abstract form (24).

## References

[1] Bers DM . 2002. Cardiac excitation-contraction coupling. Nature. 415:198–205. 11805843 10.1038/415198a

[2] Kranias EG, Hajjar RJ. 2012. Modulation of cardiac contractility by the phospholamban/SERCA2a regulatome. Circ Res. 110:1646–1660. 22679139 10.1161/CIRCRESAHA.111.259754 PMC3392125

[3] Olesen C, et al 2007. The structural basis of calcium transport by the calcium pump. Nature. 450:1036–1042. 18075584 10.1038/nature06418

[4] Bovo E, et al 2020. Dimerization of SERCA2a enhances transport rate and improves energetic efficiency in living cells. Biophys J. 119:1456–1465. 32946770 10.1016/j.bpj.2020.08.025 PMC7567987

[5] Currie S, Smith GL. 1999. Enhanced phosphorylation of phospholamban and downregulation of sarco/endoplasmic reticulum Ca2+ ATPase type 2 (SERCA 2) in cardiac sarcoplasmic reticulum from rabbits with heart failure. Cardiovasc Res. 41:135–146. 10325961 10.1016/s0008-6363(98)00241-7

[6] Haghighi K, et al 2003. Human phospholamban null results in lethal dilated cardiomyopathy revealing a critical difference between mouse and human. J Clin Invest. 111:869–876. 12639993 10.1172/JCI17892 PMC153772

[7] Kho C, et al 2011. SUMO1-dependent modulation of SERCA2a in heart failure. Nature. 477:601–605. 21900893 10.1038/nature10407 PMC3443490

[8] O'Rourke B, et al 1999. Mechanisms of altered excitation-contraction coupling in canine tachycardia-induced heart failure, I: experimental studies. Circ Res. 84:562–570. 10082478 10.1161/01.res.84.5.562

[9] Jessup M, et al 2011. Calcium upregulation by percutaneous administration of gene therapy in cardiac disease (CUPID): a phase 2 trial of intracoronary gene therapy of sarcoplasmic reticulum Ca^2+^-ATPase in patients with advanced heart failure. Circulation. 124:304–313. 21709064 10.1161/CIRCULATIONAHA.111.022889 PMC5843948

[10] Zsebo K, et al 2014. Long-term effects of AAV1/SERCA2a gene transfer in patients with severe heart failure: analysis of recurrent cardiovascular events and mortality. Circ Res. 114:101–108. 24065463 10.1161/CIRCRESAHA.113.302421

[11] Greenberg B, et al 2016. Calcium upregulation by percutaneous administration of gene therapy in patients with cardiac disease (CUPID 2): a randomised, multinational, double-blind, placebo-controlled, phase 2b trial. Lancet. 387:1178–1186. 26803443 10.1016/S0140-6736(16)00082-9

[12] Cantilina T, Sagara Y, Inesi G, Jones LR. 1993. Comparative studies of cardiac and skeletal sarcoplasmic reticulum ATPases. Effect of a phospholamban antibody on enzyme activation by Ca^2+^. J Biol Chem. 268:17018–17025. 8349590

[13] Simmerman HK, Collins JH, Theibert JL, Wegener AD, Jones LR. 1986. Sequence analysis of phospholamban. Identification of phosphorylation sites and two major structural domains. J Biol Chem. 261:13333–13341. 3759968

[14] El-Armouche A, Eschenhagen T. 2009. Beta-adrenergic stimulation and myocardial function in the failing heart. Heart Fail Rev. 14:225–241. 19110970 10.1007/s10741-008-9132-8

[15] Periasamy M, Bhupathy P, Babu GJ. 2008. Regulation of sarcoplasmic reticulum Ca^2+^ ATPase pump expression and its relevance to cardiac muscle physiology and pathology. Cardiovasc Res. 77:265–273. 18006443 10.1093/cvr/cvm056

[16] Anderson DM, et al 2016. Widespread control of calcium signaling by a family of SERCA-inhibiting micropeptides. Sci Signal. 9:ra119. 27923914 10.1126/scisignal.aaj1460 PMC5696797

[17] Bovo E, et al 2021. Presenilin 1 is a direct regulator of the cardiac sarco/endoplasmic reticulum calcium pump. Cell Calcium. 99:102468. 34517214 10.1016/j.ceca.2021.102468 PMC8541915

[18] Fisher ME, et al 2021. Dwarf open reading frame (DWORF) is a direct activator of the sarcoplasmic reticulum calcium pump SERCA. Elife. 10:e65545. 34075877 10.7554/eLife.65545 PMC8203291

[19] Shaikh SA, Sahoo SK, Periasamy M. 2016. Phospholamban and sarcolipin: are they functionally redundant or distinct regulators of the sarco(endo)plasmic reticulum calcium ATPase? J Mol Cell Cardiol. 91:81–91. 26743715 10.1016/j.yjmcc.2015.12.030 PMC4843517

[20] Torres M, Encina G, Soto C, Hetz C. 2011. Abnormal calcium homeostasis and protein folding stress at the ER: a common factor in familial and infectious prion disorders. Commun Integr Biol. 4:258–261. 21980554 10.4161/cib.4.3.15019 PMC3187882

[21] Michalak M, Robert Parker JM, Opas M. 2002. Ca^2+^ signaling and calcium binding chaperones of the endoplasmic reticulum. Cell Calcium. 32:269–278. 12543089 10.1016/s0143416002001884

[22] Gyorke S, et al 2017. The role of luminal Ca regulation in Ca signaling refractoriness and cardiac arrhythmogenesis. J Gen Physiol. 149:877–888. 28798279 10.1085/jgp.201711808 PMC5583712

[23] Bovo E, et al 2019. Novel approach for quantification of endoplasmic reticulum Ca(2+) transport. Am J Physiol Heart Circ Physiol. 316:H1323–H1331. 30901276 10.1152/ajpheart.00031.2019 PMC6620677

[24] Bovo E, Nikolaienko R, Kahn D, Espinoza-Fonseca LM, Zima AV. 2024. Regulation of SERCA2a function by the sarco/endoplasmic reticulum luminal domain. Biophys J. 123:104a.

[25] Bovo E, Martin JL, Tyryfter J, de Tombe PP, Zima AV. 2016. R-CEPIA1er as a new tool to directly measure sarcoplasmic reticulum [Ca] in ventricular myocytes. Am J Physiol Heart Circ Physiol. 311:H268–H275. 27233762 10.1152/ajpheart.00175.2016 PMC4967208

[26] Rocchetti M, et al 2005. Modulation of sarcoplasmic reticulum function by Na^+^/K^+^ pump inhibitors with different toxicity: digoxin and PST2744 [(E,Z)-3-((2-aminoethoxy)imino)androstane-6,17-dione hydrochloride]. J Pharmacol Exp Ther. 313:207–215. 15576469 10.1124/jpet.104.077933

[27] Shannon TR, Ginsburg KS, Bers DM. 2000. Reverse mode of the sarcoplasmic reticulum calcium pump and load-dependent cytosolic calcium decline in voltage-clamped cardiac ventricular myocytes. Biophys J. 78:322–333. 10620296 10.1016/S0006-3495(00)76595-7 PMC1300640

[28] Inoue M, et al 2019. Structural basis of sarco/endoplasmic Reticulum Ca(2+)-ATPase 2b regulation via transmembrane Helix interplay. Cell Rep. 27:1221–1230.e1223. 31018135 10.1016/j.celrep.2019.03.106

[29] Sitsel A, et al 2019. Structures of the heart specific SERCA2a Ca(2+)-ATPase. EMBO J. 38:e100020. 30777856 10.15252/embj.2018100020 PMC6396164

[30] Ushioda R, Nagata K. 2019. Redox-mediated regulatory mechanisms of endoplasmic reticulum homeostasis. Cold Spring Harb Perspect Biol. 11:a033910. 30396882 10.1101/cshperspect.a033910 PMC6496348

[31] Appenzeller-Herzog C . 2011. Glutathione- and non-glutathione-based oxidant control in the endoplasmic reticulum. J Cell Sci. 124:847–855. 21378306 10.1242/jcs.080895

[32] Toyoshima C, Nakasako M, Nomura H, Ogawa H. 2000. Crystal structure of the calcium pump of sarcoplasmic reticulum at 2.6 A resolution. Nature. 405:647–655. 10864315 10.1038/35015017

[33] Bublitz M, Poulsen H, Morth JP, Nissen P. 2010. In and out of the cation pumps: P-type ATPase structure revisited. Curr Opin Struct Biol. 20:431–439. 20634056 10.1016/j.sbi.2010.06.007

[34] Kekenes-Huskey PM, Metzger VT, Grant BJ, Andrew McCammon J. 2012. Calcium binding and allosteric signaling mechanisms for the sarcoplasmic reticulum Ca(2)^+^ ATPase. Protein Sci. 21:1429–1443. 22821874 10.1002/pro.2129 PMC3526986

[35] Bublitz M, et al 2013. Ion pathways in the sarcoplasmic reticulum Ca^2+^-ATPase. J Biol Chem. 288:10759–10765. 23400778 10.1074/jbc.R112.436550 PMC3624456

[36] Mekahli D, Bultynck G, Parys JB, Smedt HD, Missiaen L. 2011. Endoplasmic-reticulum calcium depletion and disease. Cold Spring Harb Perspect Biol. 3:a004317. 21441595 10.1101/cshperspect.a004317 PMC3098671

[37] Appenzeller-Herzog C, Simmen T. 2016. ER-luminal thiol/selenol-mediated regulation of Ca^2+^ signalling. Biochem Soc Trans. 44:452–459. 27068954 10.1042/BST20150233

[38] Minamino T, Kitakaze M. 2010. ER stress in cardiovascular disease. J Mol Cell Cardiol. 48:1105–1110. 19913545 10.1016/j.yjmcc.2009.10.026

[39] Glembotski CC . 2007. Endoplasmic reticulum stress in the heart. Circ Res. 101:975–984. 17991891 10.1161/CIRCRESAHA.107.161273

[40] Avezov E, et al 2013. Lifetime imaging of a fluorescent protein sensor reveals surprising stability of ER thiol redox. J Cell Biol. 201:337–349. 23589496 10.1083/jcb.201211155 PMC3628511

[41] Avezov E, et al 2015. Retarded PDI diffusion and a reductive shift in poise of the calcium depleted endoplasmic reticulum. BMC Biol. 13:2. 25575667 10.1186/s12915-014-0112-2 PMC4316587

[42] Enyedi B, Varnai P, Geiszt M. 2010. Redox state of the endoplasmic reticulum is controlled by Ero1L-alpha and intraluminal calcium. Antioxid Redox Signal. 13:721–729. 20095866 10.1089/ars.2009.2880

[43] Daiho T, et al 2001. Mutations of either or both Cys876 and Cys888 residues of sarcoplasmic reticulum Ca^2+^-ATPase result in a complete loss of Ca^2+^ transport activity without a loss of Ca^2+^-dependent ATPase activity. Role of the CYS876-CYS888 disulfide bond. J Biol Chem. 276:32771–32778. 11438520 10.1074/jbc.M101229200

[44] Ushioda R, et al 2016. Redox-assisted regulation of Ca^2+^ homeostasis in the endoplasmic reticulum by disulfide reductase ERdj5. Proc Natl Acad Sci U S A. 113:E6055–E6063. 27694578 10.1073/pnas.1605818113 PMC5068290

[45] Domeier TL, Blatter LA, Zima AV. 2009. Alteration of sarcoplasmic reticulum Ca^2+^ release termination by ryanodine receptor sensitization and in heart failure. J Physiol. 587:5197–5209. 19736296 10.1113/jphysiol.2009.177576 PMC2790258

[46] Guo T, Ai X, Shannon TR, Pogwizd SM, Bers DM. 2007. Intra-sarcoplasmic reticulum free [Ca^2+^] and buffering in arrhythmogenic failing rabbit heart. Circ Res. 101:802–810. 17704210 10.1161/CIRCRESAHA.107.152140

[47] Shannon TR, Guo T, Bers DM. 2003. Ca^2+^ scraps: local depletions of free [Ca^2+^] in cardiac sarcoplasmic reticulum during contractions leave substantial Ca^2+^ reserve. Circ Res. 93:40–45. 12791706 10.1161/01.RES.0000079967.11815.19

[48] Bovo E, et al 2024. Phosphorylation of phospholamban promotes SERCA2a activation by dwarf open reading frame (DWORF). Cell Calcium. 121:102910. 38823350 10.1016/j.ceca.2024.102910 PMC11247691

[49] NIH . 2011. *Guide for the care and use of laboratory animals*. NBK54050 [book accession]. 10.17226/12910 21595115

[50] DiNello E, et al 2020. Deletion of cardiac polycystin 2/PC2 results in increased SR calcium release and blunted adrenergic reserve. Am J Physiol Heart Circ Physiol. 319:H1021–H1035. 32946258 10.1152/ajpheart.00302.2020 PMC7789970

[51] Kuo IY, et al 2014. Decreased polycystin 2 expression alters calcium-contraction coupling and changes beta-adrenergic signaling pathways. Proc Natl Acad Sci U S A. 111:16604–16609. 25368166 10.1073/pnas.1415933111 PMC4246301

[52] Bovo E, et al 2023. Regulation of cardiac calcium signaling by newly identified calcium pump modulators. Biochem Biophys Res Commun. 685:149136. 37907012 10.1016/j.bbrc.2023.149136 PMC10841636

[53] Nikolaienko R, Bovo E, Zima AV. 2024. Expression level of cardiac ryanodine receptors dictates properties of Ca(2+)-induced Ca(2+) release. Biophys Rep (N Y). 4:100183. 39341600 10.1016/j.bpr.2024.100183 PMC11532243

[54] Cleary SR, et al 2024. Phospholamban inhibits the cardiac calcium pump by interrupting an allosteric activation pathway. J Biol Chem. 300:107267. 38583863 10.1016/j.jbc.2024.107267 PMC11098958

[55] Jo S, Kim T, Iyer VG, Im W. 2008. CHARMM-GUI: a web-based graphical user interface for CHARMM. J Comput Chem. 29:1859–1865. 18351591 10.1002/jcc.20945

[56] Salomon-Ferrer R, Gotz AW, Poole D, Grand SL, Walker RC. 2013. Routine microsecond molecular dynamics simulations with AMBER on GPUs. 2. Explicit solvent particle mesh ewald. J Chem Theory Comput. 9:3878–3888. 26592383 10.1021/ct400314y

[57] Tian C, et al 2020. ff19SB: amino-acid-specific protein backbone parameters trained against quantum mechanics energy surfaces in solution. J Chem Theory Comput. 16:528–552. 31714766 10.1021/acs.jctc.9b00591 PMC13071887

