# Supplementary material for: The endoplasmic reticulum luminal Ca2+ regulates cardiac Ca2+ pump function
Source: PNAS Nexus. 2025 Feb 7;4(2):pgaf045. doi: 10.1093/pnasnexus/pgaf045 (PMC11826342; doi:10.1093/pnasnexus/pgaf045)
Supplement: pgaf045_Supplementary_Data [file pgaf045_supplementary_data.pdf]

Supporting Information for

The endoplasmic reticulum luminal Ca^{2+} regulates cardiac Ca^{2+} pump function.

Elisa Bovo, Roman Nikolaienko, Daniel Kahn, L. Michel Espinoza-Fonseca and Aleksey V. Zima

Corresponding author: Aleksey V. Zima

Email: azima@luc.edu

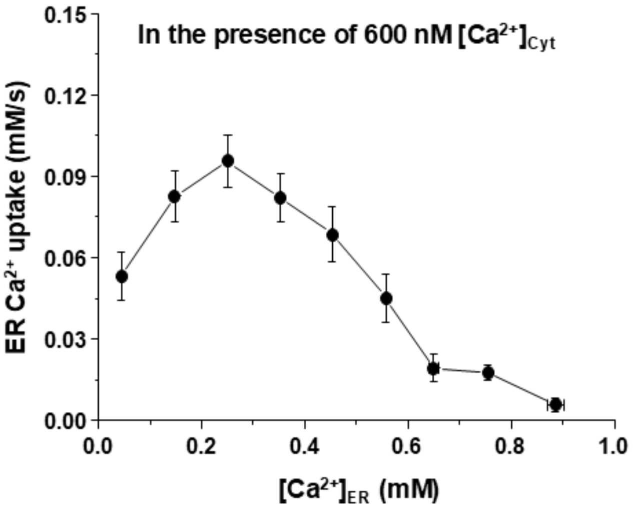

Figure S1. SERCA2a-mediated Ca^{2+} uptake as a function of $[Ca^{2+}]_{ER}$ at 600 nM $[Ca^{2+}]_{Cyt}$. For each individual cell, ER Ca^{2+} uptake was analyzed as the first derivative ($d[Ca^{2+}]_{ER}/dt$) and plotted as a function of ER Ca^{2+} load ($[Ca^{2+}]_{ER}$). The average results of SERCA2a-mediated Ca^{2+} uptake rate at different ER Ca^{2+} loads. The analysis is based on results from 15 cells.

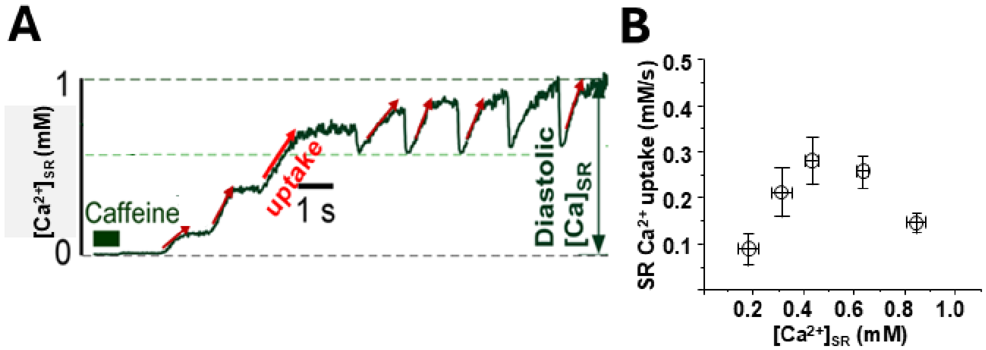

Figure S2. SERCA2a activity in intact ventricular myocytes. A. The rate of $[Ca^{2+}]_{SR}$ recovery at different SR Ca^{2+} loads in intact myocytes. SR Ca^{2+} was depleted with caffeine (5 mM) followed by electrical stimulation at 0.5 Hz. The red arrows show the rate of $[Ca^{2+}]_{SR}$ recovery after Ca^{2+} transients. **B.** The average results of SERCA-mediated Ca^{2+} uptake rate at different SR Ca^{2+} loads. The analysis is based on results from 8 myocytes.

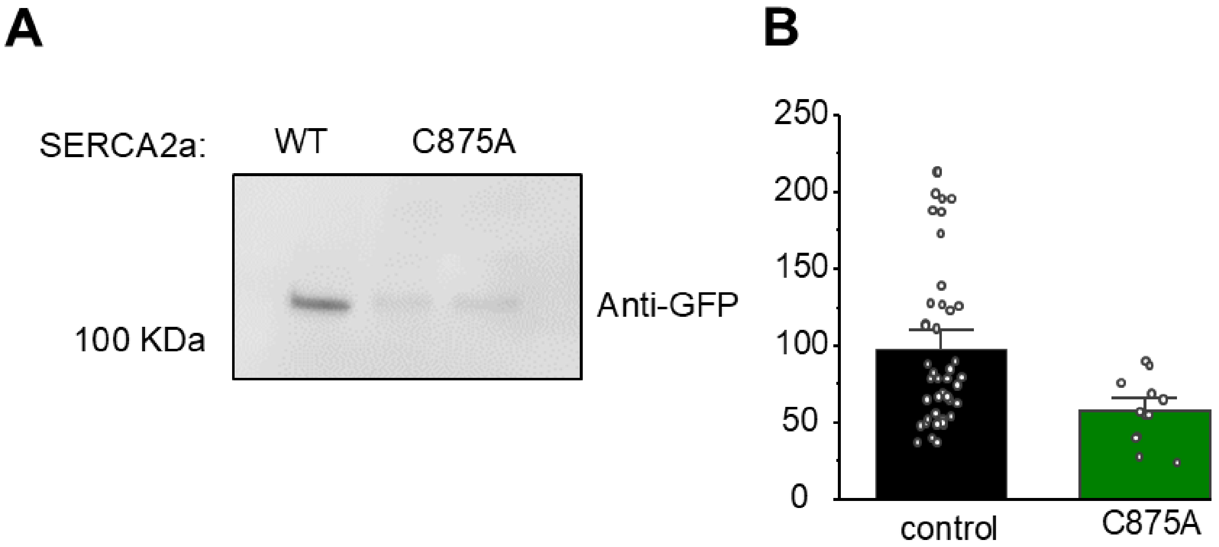

Figure S3. Western blot analysis of HEK293 cells transfected with mCer-SERCA2a (WT and the C875A mutant). A, Western blot analysis of SERCA2a expression (WT and the C875A mutant). The membrane was probed with anti-GFP antibody specific for the mCer Tag attached to SERCA2a. **B,** mCer tag fluorescence in cells expressing SERCA2a^{WT} and SERCA2a^{C875A}.

Western blot analysis

Protein collection occurred 48 hours after transfection and induction of SERCA2a expression. Cell samples were lysed in a buffer containing 1% triton and protease inhibitors. Protein quantification was performed using the Lowry based approach, DC protein assay (Bio-Rad, USA). Equal amounts of protein were run on a SDS-page and blotted on nitrocellulose using the turbo transfer system (Bio-rad, USA). Membranes were incubated with primary antibody anti-SERCA2 (IID8, Santa Cruz, USA) and developed using the HRP conjugated secondary antibody(1). Western blots were imaged in a ChemiDoc (Bio-rad, USA) apparatus after incubation with the HRP chemiluminescent substrate (Millipore, USA).

Reference List

1. E. Bovo *et al.*, Dimerization of SERCA2a Enhances Transport Rate and Improves Energetic Efficiency in Living Cells. *Biophys J* **119**, 1456-1465 (2020).
